# Production of fuels and chemicals from xylose by engineered *Saccharomyces cerevisiae*: a review and perspective

**DOI:** 10.1186/s12934-017-0694-9

**Published:** 2017-05-11

**Authors:** Suryang Kwak, Yong-Su Jin

**Affiliations:** Department of Food Science and Human Nutrition and Carl R. Woose Institute for Genomic Biology, University of Illinois at Urbana-Champaign, Urbana, IL USA

**Keywords:** Xylose, *Saccharomyces cerevisiae*, Metabolic engineering

## Abstract

Efficient xylose utilization is one of the most important pre-requisites for developing an economic microbial conversion process of terrestrial lignocellulosic biomass into biofuels and biochemicals. A robust ethanol producing yeast *Saccharomyces cerevisiae* has been engineered with heterologous xylose assimilation pathways. A two-step oxidoreductase pathway consisting of NAD(P)H-linked xylose reductase and NAD^+^-linked xylitol dehydrogenase, and one-step isomerase pathway using xylose isomerase have been employed to enable xylose assimilation in engineered *S. cerevisiae*. However, the resulting engineered yeast exhibited inefficient and slow xylose fermentation. In order to improve the yield and productivity of xylose fermentation, expression levels of xylose assimilation pathway enzymes and their kinetic properties have been optimized, and additional optimizations of endogenous or heterologous metabolisms have been achieved. These efforts have led to the development of engineered yeast strains ready for the commercialization of cellulosic bioethanol. Interestingly, xylose metabolism by engineered yeast was preferably respiratory rather than fermentative as in glucose metabolism, suggesting that xylose can serve as a desirable carbon source capable of bypassing metabolic barriers exerted by glucose repression. Accordingly, engineered yeasts showed superior production of valuable metabolites derived from cytosolic acetyl-CoA and pyruvate, such as 1-hexadecanol and lactic acid, when the xylose assimilation pathway and target synthetic pathways were optimized in an adequate manner. While xylose has been regarded as a sugar to be utilized because it is present in cellulosic hydrolysates, potential benefits of using xylose instead of glucose for yeast-based biotechnological processes need to be realized.

## Background

With rising energy demand and environmental pollution, searching for promising alternative energy sources replacing conventional non-renewable fossil fuels is imperative. Microbial conversion of renewable biomass, such as plant cell walls, into biofuels and chemicals is a plausible option to substitute petroleum refineries in a sustainable manner. To develop economic and sustainable conversion processes at an industrial scale, substrates of the microbial conversion must be cheap, eco-friendly, and not competing with food supply to avoid ethical issues [1]. Lignocellulosic biomass, such as energy crops and agricultural residues, is an ideal candidate as renewable source satisfying the conditions. Glucose and xylose are most abundant monosaccharides in lignocellulosic biomass taking up 60–70 and 30–40% of their hydrolysates, respectively [1, 2]. Thus efficient xylose-fermenting microbial strains are essential for developing economically feasible bioconversion processes using the renewable biomasses.

Although there are many bacterial and yeast strains capable of naturally utilizing xylose, *Saccharomyces cerevisiae* has advantages over the innate xylose-utilizing microorganisms regarding robustness against various stresses in industrial environments, such as low pH, high osmotic pressure, high alcohol concentration, and phage contamination [3–5]. Fundamental strategies to construct efficient xylose-fermenting yeasts using *S. cerevisiae* and yeast co-fermentation of mixed sugars in lignocellulosic hydrolysates have been previously reviewed in depth [2, 6–10]. In this review, therefore, we focus more on the utilization of xylose as a sole carbon source by engineered yeast, and intend to discuss recent advances in metabolic engineering studies to overcome limitations on xylose fermentation by engineered yeast. Additionally, we demonstrate distinct features in metabolic physiology during xylose fermentation by engineered yeast, and potential benefits of xylose as a carbon source to produce other valuable fuels and chemicals instead of ethanol [11, 12].

## Yeast xylose metabolism

**Fig. 1:**
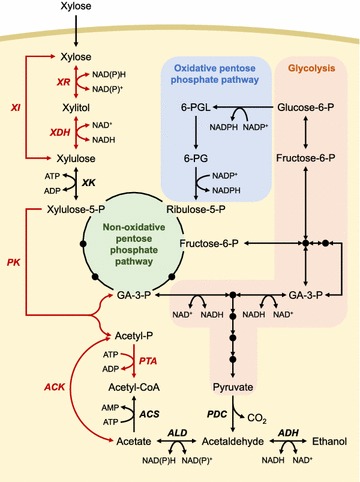
Overview of xylose assimilation pathways in yeasts. *Red items* indicate heterologous metabolic pathways which have been introduced into *S. cerevisiae*. *6-PGL* 6-phosphoglucono-1,5-lactone, *6-PG* 6-phosphogluconate, *GA-3-P* glyceraldehyde-3-phosphate

Xylose assimilation requires the isomerization of xylose into xylulose and subsequent phosphorylation of xylulose into xylulose-5-phosphate which is an inlet metabolite to pentose phosphate pathway. Xylose-fermenting microorganisms engage two distinct pathways, the oxidoreductase pathway and the isomerase pathway, for conversion of xylose into xylulose (Fig. 1). Xylose-fermenting yeasts employ the oxidoreductase pathway consisting of two enzymatic reactions of xylose reductase (XR, EC 1.1.1.30) and xylitol dehydrogenase (XDH, EC 1.1.1.9), which convert xylose to xylulose via xylitol. The xylose-fermenting yeast strains assimilate xylose mainly under aerobic conditions. XR has a dual cofactor preference with NADPH and NADH, whereas XDH uses only NAD^+^. On the other hand, the isomerase pathway consists of one enzymatic reaction of xylose isomerase (XI, EC 5.3.1.5). XI catalyzes various sugar interconversions of aldose and ketose including xylose and xylulose without cofactor requirement. Although most XIs have been identified from bacterial strains [13], anaerobic fungi assimilating xylose via XI, such as *Piromyces* [14] and *Orpinomyces* [15], also have been discovered. Xylulokinase (XK, EC 2.7.1.17) catalyzes the xylulose phosphorylation to xylulose-5-phosphate, an intermediate of the non-oxidative pentose phosphate pathway and the phosphoketolase pathway.

The pentose phosphate pathway is a universal metabolic pathway using xylulose-5-phosphate as a metabolic intermediate. It can be divided into two distinct phases, an oxidative phase and a non-oxidative phase. Yeast cells metabolize xylulose-5-phosphate through the non-oxidative pentose phosphate pathway and form various phosphorylated sugars of three, four, five, six, and seven carbons which serve as intermediates of glycolysis or precursors of cell components such as nucleotides and amino acids [16]. The oxidative pentose phosphate pathway is a major route for generating NADPH which functions as a driving force for the XR activity and a protection mechanism against oxidative stresses [16, 17] (Fig. 1).

Xylulose-5-phosphate can also be metabolized through the action of phosphoketolase (PK, EC 4.1.2.9) in several xylose-fermenting yeasts including *Candida tropicalis*, *Rhodotorula graminis*, and *Rhodotorula glutinis* [18], although PK pathway is more common in prokaryotes [19]. PK cleaves xylulose-5-phosphate to form glceraldehyde-3-phosphate and acetyl phosphate. While glyceraldehyde-3-phosphate can be further metabolized through glycolysis and non-oxidative pentose phosphate pathway (Fig. 1), the metabolism of acetyl phosphate in these xylose-fermenting yeasts has not been clearly disclosed up to now. Some other eukaryotic microorganisms can convert acetyl phosphate into acetyl-Coenzyme A (acetyl-CoA) through combined reactions of acetate kinase (ACK, EC 2.7.2.1) and acetyl-CoA synthase (ACS, EC 6.2.1.1) or a single reaction of phosphotransacetylase (PTA, EC 2.3.1.8) [20] (Fig. 1). Recently, Meadows et al. [21] reported that yeast glycerol-3-phosphate phosphatases have a promiscuous phosphatase activity on acetyl phosphate, resulting in acetate formation from acetyl phosphate.

## Construction of efficient xylose-fermenting *S. cerevisiae* for ethanol production

Despite the abundance of xylose in nature, only a few yeasts naturally have the ability to assimilate xylose [22, 23]. Interestingly, many yeasts possess the genes coding for XR, XDH, and XK, yet a considerable number of the yeasts do not grow on xylose or shows varied xylose-fermenting abilities among strains. It reflects other possible factors allowing yeasts to metabolize xylose beyond presence or absence of the xylose pathway, such as regulations of xylose pathway expression or characteristics of enzymes [24]. Among the innate xylose-fermenting yeasts, *Scheffersomyces stipitis* is one of the most studied yeasts regarding its biochemistry of xylose pathway and xylose conversion into ethanol [23, 25]. For heterologous xylose pathway expression in the eukaryotic host system, *Sc. stipitis* genes encoding xylose pathway have been more widely used than any other eukaryotic xylose pathway [6–9]. *Spathaspora passalidarum* is a relatively recently identified beetle-associated yeast [26] and currently the most promising native xylose-fermenting yeast in terms of growth and ethanol fermentation on xylose [27, 28]. *Sp. passalidarum* has NADH-preferred XR that allows *Sp. passalidarum* to more efficiently consume xylose and produce ethanol under both aerobic and anaerobic culture conditions as compared to *Sc. stipitis* and other native xylose-fermenting yeasts [27]. However, their tolerances against ethanol and inhibitors in lignocellulosic hydrolysates, such as furfural, hydroxymethyfurfural, and organic acids, are low [29, 30], and the pefromance of xylose fermentation by the native xylose-fermenting yeast strains strongly depends on culture conditions [31].

Consequently, various metabolic engineering approaches have been undertaken to introduce xylose metabolic pathways into *S. cerevisiae*—the best genetically characterized yeast as the first eukaryote whose whole genome was completely sequenced [32]—to build economically feasible xylose fermentation processes. *S. cerevisiae* is an indispensable microbial cell factory in current biotechnology and food industries, especially as an ethanol producer, due to its high fermentation rate and robustness to environmental stresses including tolerance to alcoholic products [3–5]. However, *S. cerevisiae* cannot naturally utilize xylose, while it can assimilate xylulose through endogenous xylulokinase (*XKS1*) and pentose phosphate pathway [33]. Metabolic engineering approaches for introducing heterologous xylose utilization pathways and optimizing internal metabolisms have been undertaken to develop efficient xylose-fermenting *S. cerevisiae* strains. Performances of representative xylose-fermenting *S. cerevisiae* strains were compared in Table 1.

**Table 1:** Comparison of performances of representative xylose-fermenting engineered *S. cerevisiae*

Strains	Strain descriptions		Culture conditions	Xylose consumption rate	Ethanol production rate	Ethanol yield (g/g xylose)	Reference
H131-A3-AL^CS^	XI (*Piromyces XYLA*)	*SsXYL3*, *SsTAL1, TKL1, RPE1, RKI1*	Anaerobic batch, 2× YNB, 4% xylose	1.866 (g g^−1^ h^−1^)	0.765 (g g^−1^ h^−1^)	0.410	[44]
SXA-R2P-E	XI (mutant *Piromyces XYLA*)	*SsTAL1, Δgre3, Δpho13*	Anaerobic batch, YNB, 4% xylose	0.077(g OD^−1^ h^−1^)	0.033 (g OD^−1^ h^−1^)	0.453	[61, 95]
SR8	XR–XDH (*SsXYL1*, *SsXYL2*)	*SsXYL3*, *Δald6*, null mutant of *PHO13*	Anaerobic batch, YNB, 4% xylose	0.129 (g OD^−1^ h^−1^)	0.046 (g OD^−1^ h^−1^)	0.378	[57, 61]
TMB 3422	XR–XDH (mutant *SsXYL1*, *SsXYL2*)	*XKS1, TAL1, TKL1, RPE1, RKI1, Δgre3*	Anaerobic batch, 2× YNB, 5% xylose	0.580 (g g^−1^ h^−1^)	0.180 (g g^−1^ h^−1^)	0.340	[74, 150]
TMB 3504	XR–XDH (*SpXYL1.2*, *SsXYL2*)	*XKS1, TAL1, TKL1, RPE1, RKI1, Δgre3*	Anaerobic batch, 2× YNB, 5% xylose	0.760 (g g^−1^ h^−1^)	0.330 (g g^−1^ h^−1^)	0.400	[74]

*Ss Sc. stipitis*, *Sp Sp. passalidarum*

### Engineering with the xylose isomerase pathway

XI coding genes are mainly spread over bacterial genomes. Accordingly, initial attempts to introduce the XI pathway into *S. cerevisiae* employed bacterial XI genes. However, most of them were unsuccessful due to the difficulties in expressing bacterial XIs functionally in yeast [34–36]. There was an approach searching novel XIs from soil metagenomics library based on protein sequences and activities in *Escherichia coli*, yet the identified XIs could not perform in *S. cerevisiae* as strong as in *E. coli* [37]. Discovery and application of eukaryotic XI coding genes from anaerobic fungi [14, 15, 38–40], and bacterial XI genes of *Thermus thermophiles* [41], *Clostridium phytofermentans* [42], and *Bacteroides stercoris* [43] which can be functionally expressed in *S. cerevisiae* enabled successful xylose fermentation by engineered *S. cerevisiae*.

Artificial adaptation of heterologous XIs to enhance expression and activity in *S. cerevisiae* has been conducted to improve xylose fermentation by XI expressing *S. cerevisiae*. First, codon optimization and increased gene dosages of XI can improve XI activity in *S. cerevisiae* [42, 44]. For example, Brat et al. changed the *C. phytofermentans* XI coding sequence based on the codon usage of glycolytic pathway genes which are highly expressed in *S. cerevisiae* [45]. The codon optimization enhanced the XI activity in *S. cerevisiae*, resulting in a 46% increase in specific growth rate on xylose [42]. Laboratory evolution of a XI expressing *S. cerevisiae* led to amplification of an expression cassette containing a codon-optimized XI [44, 46]. The higher copy number of the XI expression cassette resulted in a higher transcriptional level and consequently enhanced the XI enzymatic activity of the evolved *S. cerevisiae* compared to the parental strain containing a single copy of the codon-optimized XI [44, 46].

Second, directed evolution is also an effective strategy to improve kinetic properties of XI expressed in *S. cerevisiae*. Lee et al. [47] screened XI mutants which enable higher growth rate of the transformant *S. cerevisiae* on xylose, after introducing mutated *XYLA* from *Piromyces* sp. via three rounds of mutagenesis and growth-based screening under xylose conditions. Six amino acid substitutions (E15D, E144G, E129D, A177T, T242S, and V433I) were accumulated in the final mutant *XYLA* during the three rounds of directed evolution. The six-amino acid-mutated XI exhibited a 77% higher *V*_*max*_ as compared to the wildtype. The essential and synergistic mutations, E15D and T242S, were further identified among the six mutations of the final mutant. Expression of the mutant XI (E15D and T242S) resulted in 8 times higher ethanol production and xylose consumption rate of *S. cerevisiae* as compared to wildtype *Piromyces* XI expression [47].

Recent two studies on laboratory evolution of XI expressing *S. cerevisiae* strains have reported novel engineering targets, which are not directly related to sugar catabolism, to improve xylose utilization. Both laboratory evolution studies with bacterial XI (*C. phytofermentans xylA*) under aerobic conditions [48], and fungal XI (*Orpinomyces* sp. *XYLA*) under semi-anaerobic conditions [46] discovered that loss-of-function mutations of *ISU1*, which encodes a conserved mitochondrial matrix protein participating in assembly of iron-sulfur cluster, enhanced xylose fermentation of the XI expressing *S. cerevisiae* strains. Laboratory evolution of the fungal XI expressing *S. cerevisiae* discovered an additional loss-of-function mutation of *SSK2* which further enhanced xylose assimilation of the *isu1* mutant *S. cerevisiae* [46]. Ssk2p positively interacts with HOG (high-osmolarity glycerol) pathway, which is known to be involved in the cation homeostasis, by direct phosphorylation of Hog1p [49]. Interestingly, laboratory evolution of the bacterial XI expressing *S. cerevisiae* observed beneficial effects of loss of *HOG1* function regarding xylose fermentation under aerobic conditions [48]. Loss-of-function mutations on *ISU1*, *SSK2*, and *HOG1* could cause impaired biosynthesis of the iron–sulfur cluster and increased the availability of iron ion [46, 48, 50]. Higher availability of iron ion (Fe^2+^) may activate metalloenzyme XI [51] or promote aerobic sugar catabolism via biosynthesis of iron-containing heme and formation of active cytochrome C oxidase subunits. Loss of *ISU1* function indeed led to the enhanced respiratory metabolism of mitochondria in the XI expressing *S. cerevisiae* on xylose [48]. The model of the iron availability was supported by the improved xylose assimilation of the XI expressing *S. cerevisiae* through iron supplementation [46].

As Hog1p up-regulates transcriptional levels of *GRE3* coding for aldose reductase involved in the formation of xylitol [48, 52], loss of *HOG1* function also could indirectly enhance xylose assimilation in a XI expressing *S. cerevisiae* due to the reduction of xylitol production [48]. Additional laboratory evolution under anaerobic conditions resulted in the direct loss of *GRE3* function in the evolved *S. cerevisiae*, indicating that reduced expression levels of *GRE3* by the *hog1* mutation might not be adequate during anaerobic xylose fermentation [48]. The laboratory evolution under anaerobic conditions also resulted in a loss-of-function mutation in *IRA2*, which encodes an inhibitor reducing cAMP levels [48, 53]. As the increase in cAMP concentration activates key glycolytic enzyme reactions via cAMP-protein kinase A pathway [54], the *ira2* mutation may improve xylose consumption and ethanol production of engineered *S. cerevisiae* by enhancing glycolytic flux. Interestingly, the positive effects of the *ira2* mutation during anaerobic xylose assimilation required the *isu1* mutation [48].

### Engineering with the xylose reductase–xylitol dehydrogenase pathway

Most metabolic engineering studies expressing heterologous XR–XDH pathway in *S. cerevisiae* mainly employed *Sc. stipitis XYL1* and *XYL2* genes coding for XR and XDH, respectively, to fill the metabolic gap between xylose and xylulose of *S. cerevisiae* [55–59]. Engineered *S. cerevisiae* with the XR–XDH pathway exhibited faster xylose assimilation rate and higher ethanol titer than engineered *S. cerevisiae* with the XI pathway [60, 61]. However, the XR–XDH pathway has a drawback of the cofactor imbalance between the XR (mainly NADPH-dependent) and XDH (NAD^+^-dependent), especially under anaerobic conditions where NADH cannot be oxidized to NAD^+^ using oxygen as an electron acceptor. The different cofactor preferences of XR and XDH can result in NAD^+^ deficiency (or surplus NADH) which may cause the accumulation of xylitol under anaerobic conditions. As such, engineered yeast with the XR–XDH pathway exhibited lower ethanol yields than engineered yeast with the XI pathway [60, 61].

Dynamic flux balance analysis predicted faster xylose assimilation and higher ethanol titers by engineered *S. cerevisiae* expressing cofactor-balanced oxidoreductase pathway [62]. Also, protein engineering approaches to alter cofactor preferences of XR to NADH [63–66] or of XDH to NADP^+^ [67, 68] through protein structure analysis and site-directed mutagenesis of cofactor binding pockets have been reported. It was demonstrated that combinations of NADH-specific mutant XR and wild-type XDH [69–71] or wild-type XR and NADP^+^-specific mutant XDH [72, 73] reduce xylitol accumulation and improve ethanol production from xylose as compared to *S. cerevisiae* expressing the wild-type *Sc. stipitis* XR and XDH. Similarly, replacement of *Sc. stipitis* XR by NADH-preferred XR from *Sp. passalidarum* (*XYL1.2*) increased ethanol yield and productivity of engineered *S. cerevisiae* engineered with *Sc. stipitis* XR–XDH pathway [74] (Table 1).

Increasing XDH activity compared to XR activity in engineered *S. cerevisiae* can be an alternative approach to reducing xylitol accumulation. Eliasson et al. simulated xylose assimilation of engineered *S. cerevisiae* using a simplified kinetic model of XR–XDH–XK reactions and predicted that xylitol accumulation would be minimized when the activity ratio of XR/XDH is less than 0.1 [75]. Also, engineered *S. cerevisiae* with low XR/XDH ratios led to reduced xylitol accumulation and higher ethanol yield [75, 76]. Similarly, the efficiency of the oxidoreductase pathway can be improved by increasing XDH activities when XR activity levels are fixed [77–79].

**Fig. 2:**
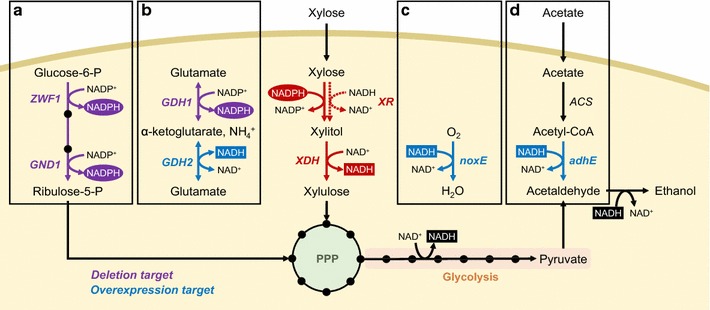
Control of redox balance by manipulating endogenous metabolism (**a**, **b**) and furnishing heterologous electron sink reactions (**c**, **d**). Endogenous NADPH regenerating enzymes of the oxidative pentose phosphate pathway (**a**) and the ammonia utilization pathway were deleted to reduce XR activity and accordingly develop a lower XR/XDH activity ratio. Surplus NADH was alleviated via NADH oxidase reaction (**a**) or utilized as a driving force of acetate reduction pathway consisting of acetyl-CoA synthase (ACS) and acetylating acetaldehyde dehydrogenase (AADH) (**b**). Gene nomenclature: *ZWF1* glucose-6-phsophate dehydrogenase, *GND1* 6-phosphogluconate dehydrogenase, *GDH1* NADPH-dependent glutamate dehydrogenase, *GDH2* NADH-dependent glutamate dehydrogenase, *noxE* NADH oxidase from *L. lactis, adhE* AADH from *Escherichia coli*

Instead of altering expression levels of XR and XDH, some studies manipulated endogenous oxidoreductase pathways in *S. cerevisiae* to change NADH/NADPH ratio so that XR and XDH reaction can be operated without causing redox imbalance. As the oxidative phase of pentose phosphate pathway is a major metabolic route for generating NADPH in *S. cerevisiae* (Fig. 1), knockout of related genes such as *ZWF1* (glucose-6-phosphate dehydrogenase, EC 1.1.1.49) and *GND1* (6-phosphogluconate dehydrogenase, EC 1.1.1.44) substantially decreased cellular NADPH levels, resulting in lower XR/XDH activity ratios and lower xylitol yield from xylose [80, 81] (Fig. 2a). However, the impaired XR activities due to reduced NADPH levels in the *Δzwf1* and *Δgnd1* mutants resulted in poor xylose fermentation and growth inhibition even though xylitol accumulation reduced substantially. Verho et al. attempted to alleviate the negative effect of *ZWF1* deletion by introducing a heterologous NADP^+^-dependent glyceraldehyde-3-phosphate dehydrogenase (*Kluyveromyces lactis GPD1*, EC 1.2.1.13). Through the combination of *ZWF1* deletion and *KlGPD1* overexpression, they achieved 48% less xylitol yield and 52% higher ethanol titer compared to parental *S. cerevisiae* expressing the XR–XDH pathway [81]. Similarly, deletion of *GDH1* encoding NADPH-dependent glutamate dehydrogenase (EC 1.4.1.4) and the overexpression of *GDH2* encoding NADH-dependent glutamate dehydrogenase (EC 1.4.1.2) in the ammonia utilization pathway reduced xylitol accumulation and increased ethanol yield during xylose fermentation [82] (Fig. 2b). Additionally, deletion of NADP^+^-dependent cytosolic aldehyde dehydrogenase (*ALD6*, EC 1.2.1.4) improved xylose fermentation and reduced accumulation of acetate [57, 70].

The introduction of heterologous electron sink reactions capable of oxidizing surplus NADH was able to alleviate the cofactor imbalance and xylitol accumulation of engineered yeast with the oxidoreductase pathway. For example, overexpression of *noxE* encoding a water-forming NADH oxidase (EC 1.6.99.3) from *Lactococcus lactis* in engineered yeast with the XR–XDH pathway resulted in a 70% decrease of xylitol yield and 39% increase of ethanol yield through regenerating NAD^+^ from NADH with molecular oxygen as an electron acceptor (Fig. 2c) [83]. Instead of the water-forming reaction, Wei et al. constructed the acetate reduction pathway consisting of ACS and acetylating acetaldehyde dehydrogenase (AADH, EC 1.2.1.10) which converts acetyl-CoA into acetaldehyde (Fig. 2d) [84]. Acetate is one of the major byproducts in lignocellulosic hydrolysates, and it hampers fermentation and growth of yeast cells in lignocellulosic hydrolysates [85]. The acetate reduction pathway can exploit the cofactor imbalance of the XR–XDH pathway as surplus NADH is necessary to reduce acetyl-CoA into ethanol. The efficiency of the acetate reduction pathway depended upon expression levels and activities of ACS and AADH [86]. This acetate utilization strategy accomplished efficient xylose fermentation—less xylitol accumulation and more ethanol production—by utilizing the non-carbohydrate component acetate, and consequently detoxifying lignocellulosic hydrolysates [84, 86].

Similarly, re-assimilation of carbon dioxide through reductive pentose phosphate pathway consisting of carboxylase/oxygenase (RuBisCO, EC 4.1.1.39) and phosphoribulokinase (PRK, EC 2.7.1.19) also can be used as an electron sink reaction. Serial reactions of PRK and RuBisCO synthesize two molecules of 3-phosphoglycerate from ribulose-5-phosphate and carbon dioxide. As 3-phosphoglycerate eventually can be converted into ethanol, carbon dioxide can act as an electron acceptor for the re-oxidation of NADH [87, 88]. Xia et al. [87] constructed functional reductive pentose phosphate pathway in an efficient xylose-fermenting *S. cerevisiae* by introducing RuBisCo, PRK, and chaperonins to re-assimilate carbon dioxide generated by pyruvate decarboxylase (PDC, EC 4.1.1.1) during ethanol fermentation and consequently increase ethanol yield. The functional expression of reductive pentose phosphate pathway in engineered *S. cerevisiae* distinctly reduced yields of glycerol and xylose, which were formed to compensate the excessive NADH, and increased ethanol yield during xylose fermentation, whereas it did not significantly change glycerol and ethanol yields of glucose fermentation [87, 88]. The synergistic effect of xylose utilization and carbon dioxide re-assimilation resulted from the better supply of ribulose-5-phosphate and the excessive NADH during xylose fermentation than glucose fermentation [87]. On the other side, NADPH and ATP supply for XR and RuBisCO, respectively, could be limited during xylose fermentation of engineered *S. cerevisiae*. Recently, Li et al. [89] have demonstrated and solved the issue of NADPH and ATP limitation during carbon dioxide re-assimilation under xylose culture conditions through co-fermentation of maltose and xylose.

### Reconfiguration of endogenous sugar metabolism

XK plays an important role as a linker reaction between foreign xylose assimilation pathways and native pentose phosphate pathway in engineered *S. cerevisiae*. Interestingly, *S. cerevisiae* shows a bigger gap in the growth rates between glucose and xylulose as a sole carbon source than other yeast strains [33], suggesting that XK activity needs to be enhanced along with XR–XDH [57, 75, 90–94] or XI [15, 38, 44, 95–97] to rapidly ferment xylose by engineered *S. cerevisiae*. Additional expression of endogenous XK coding gene *XKS1* under the control of a strong promoter along with *XYL1* and *XYL2* improved xylose fermentation drastically [55], yet *Sc. stipitis* XK (*XYL3*) also has been adopted due to its higher activity and narrower specificity than endogenous XK [98, 99]. Increase in XK activities improved xylulose fermenting capability of *S. cerevisiae* [33, 99, 100], reduced xylitol accumulation, and improved ethanol production in xylose-fermenting *S. cerevisiae* [55, 90–93, 96]. At the same time, however, excessive expression of XK is toxic to the yeast cells under xylulose [101] or xylose culture conditions [98, 102]. As XK requires ATP as a substrate, overexpression of XK with an uncontrolled supply of xylulose can result in an imbalance between ATP consumption and ATP generation, and eventually ATP depletion [98]. The toxic effect of XK overexpression during xylose fermentation can be alleviated by the disruption of *PHO13* [102] or moderated XK activity levels [93, 98]. These studies indicate that the low XK activity of *S. cerevisiae* is one of the rate-limiting steps in the fermentation of xylulose and xylose, yet its expression level should be carefully adjusted with consideration of the activities of upstream and downstream metabolic pathways.

**Fig. 3:**
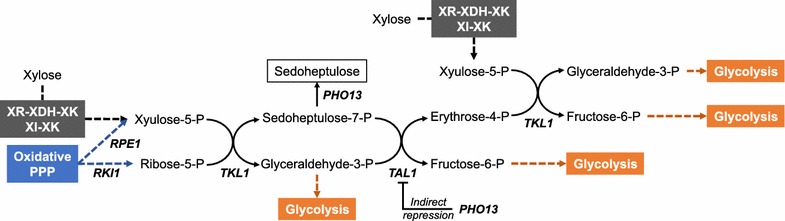
Schematics of the non-oxidative pentose phosphate pathway. While other intermediates can be shunted to glycolysis, sedoheptulose-7-phosphate is converted into a dead-end metabolite sedoheptulose by promiscuous phosphatase activity of Pho13p. The expression of *TAL1* is indirectly regulated by *PHO13*. Gene nomenclature: *RKI1* ribose-5-phosphate isomerase, *RPE1* ribulose-5-phosphate epimerase, *TKL1* transketolase, *TAL1* transaldolase

Beyond optimization of xylose isomerization and xylulose phosphorylation pathways, additional genetic perturbations for eliciting improved xylose fermentation have been identified through rational and inverse metabolic engineering approaches. Non-oxidative pentose phosphate pathway was hypothesized as a limiting pathway of engineered *S. cerevisiae* for xylose fermentation as intermediates of the non-oxidative pentose phosphate pathway were accumulated under xylose culture conditions [58, 103]. To overcome the inefficient capacity of the non-oxidative pentose phosphate pathway in *S. cerevisiae*, endogenous or *Sc. stipitis* genes coding for the enzymes involved in the non-oxidative pentose phosphate pathway, such as *RKI1* (ribose-5-phosphate isomerase, EC 5.3.1.6), *RPE1* (ribulose-5-phosphate epimerase; EC 5.1.3.1), *TKL1* (transketolase, EC 2.2.1.1), and *TAL1* (transaldolase, EC 2.2.1.2) (Fig. 3), have been overexpressed. Overexpression of the enzymes involved in non-oxidative pentose phosphate pathway led to improved xylose assimilation rates by engineered *S. cerevisiae* expressing the XR–XDH pathway [59, 70, 104–106] or the XI pathway [38, 44, 107].

Inverse metabolic engineering studies have discovered various overexpression and knockout targets which are hidden limiting reactions and unknown coupled regulating factors of xylose utilization of *S. cerevisiae*. Among those targets, *PHO13* has been consistently identified as one of the most effective deletion targets to enhance xylose-fermenting capabilities of *S. cerevisiae* [57, 102]. Under xylose conditions, deletion of *PHO13* improves the cell growth rate and the ethanol productivity of engineered *S. cerevisiae* strains expressing the XR–XDH pathway [57, 108, 109] or XI pathway [95] when activities of heterologous xylose pathways reach sufficient levels. Although physiological functions of Pho13p have not been fully disclosed yet, a recent study found significant changes in transcriptional patterns of *S. cerevisiae* after *PHO13* deletion, in particular, the oxidative pentose phosphate pathway, other NADPH-regenerating pathways, and *TAL1* encoding transaldolase [110]. Transaldolase is a bottleneck of the non-oxidative pentose phosphate pathway resulting in the accumulation of its substrate sedoheptulose-7-phosphate and sedoheptulose, a dead-end metabolite, during xylose fermentation in *S. cerevisiae*. Interestingly, Pho13p has a promiscuous phosphatase activity on sedoheptulose-7-phosphate. Thus *PHO13* deletion significantly enhanced xylose fermentation and reduced accumulation of sedoheptulose-7-phosphate and sedoheptulose (Fig. 3). These phenotypic changes of the *PHO13* deleted strain could be similarly reproduced by single overexpression of *TAL1* [106].

### Introduction of the phosphoketolase pathway

**Fig. 4:**
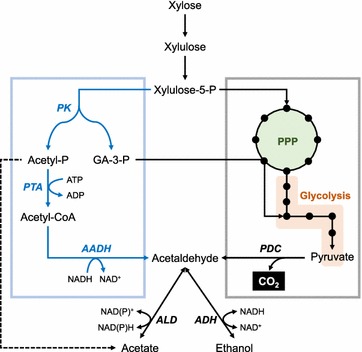
Comparison of a synthetic PK-PTA-AADH pathway (*left*, *blue*) and native metabolism of xylulose-5-phosphate (*right*) via pentose phosphate pathway and glycolysis. The synthetic pathway is more carbon-conserving in ethanol production, compared to native metabolism, due to its absence of carbon losing enzymatic reactions, such as pyruvate decarboxylase. The *dotted arrow* indicates promiscuous acetyl phosphate phosphatase activities of *S. cerevisiae*

*S. cerevisiae* and other yeast strains possessing neither PK nor ACK/PTA pathways metabolize xylulose-5-phosphate only through pentose phosphate pathway and glycolysis. This oxidative glycolysis of sugars inevitably loses one carbon via carbon dioxide generation during conversion of pyruvate to acetaldehyde which can be further metabolized to acetate or ethanol (Fig. 4). As the combination of PK and ACK/PTA theoretically converts xylulose-5-phosphate to acetate or acetyl-CoA with glyceraldehyde-3-phosphate without carbon loss (Figs. 1, 4), several studies attempted to increase the yield of target molecules by introducing heterologous PK into *S. cerevisiae*. While the introduction of PK and ACK/PTA enhanced production of derivatives of cytosolic acetyl-CoA such as fatty acid ethyl esters and polyhydroxy butyrate under glucose culture conditions [111, 112], the performance of PK under xylose culture conditions is still ambiguous. Sonderegger et al. [113] attempted to increase ethanol yield on xylose through the carbon conserving route of PK, PTA, and AADH (Fig. 4). Overexpression of PK in *S. cerevisiae*, however, resulted in significant accumulation of acetate under xylose culture conditions, instead of ethanol. Interestingly, they argued that *S. cerevisiae* showed marginal, yet reliable endogenous activities of PK. Overexpression of PTA and AADH without furnishing heterologous PK resulted in 25% increase in ethanol yield on xylose. Given the potential of the carbon conserving nature of the PK pathways, in-depth investigation of the innate PK activity in *S. cerevisiae* will be necessary.

### Xylose transporter engineering

As *S. cerevisiae* does not have xylose-specific transporters, xylose-fermenting *S. cerevisiae* strains might consume xylose via native hexose transporters, such as Hxt1p, Hxt2p, Hxt4p, Hxt5p, Hxt7p, and Gal2p [114–116]. However, the characteristics of non-specific sugar transporters, such as much less affinity toward xylose than glucose [117], and inefficient transport at lower xylose concentrations [55, 118], can be another bottleneck to construct an efficient xylose-fermenting yeast for industrial applications. Mainly two strategies—search for novel heterologous xylose transporters, and engineering of existing sugar transporters—have been employed to overcome the limited xylose-transporting capacity of native sugar transporters in *S. cerevisiae*.

Xylose-transporting capabilities of heterologous transporters from innate xylose-fermenting yeasts, such as *Candida intermedia* and *Sc. Stipitis*, have been examined in *S. cerevisiae*. The heterologous expression of *C. intermedia GXF1* and *GXS1*, *Sc. stipitis XUT1* and *XUT2* in hexose transporter null mutant *S. cerevisiae* (*Δhxt1*–*17*, *Δgal2*, *Δstl1*, *Δagt1*, *Δmph2*, and *Δmph3*) marginally affected growth as compared to the expression of endogenous transporters, such as Hxt7p [119], while additional expression of *GXF1* in transporter-positive *S. cerevisiae* notedly improved xylose uptake rate and cell growth in low xylose concentration [120]. Additional expression of *Sc. stipitis* transporters, such as *XUT4*, *XUT5*, *XUT6*, *XUT7*, *RGT2*, and *SUT4*, also increased xylose uptake rates and specific growth rates of industrial *S. cerevisiae* harboring XI [121].

Young et al. generated mutant xylose transporters through directed evolution of *C. intermedia GXS1* and *Sc. stipitis XUT3*. Introduction of the mutant transporters (*gxs1-2.1* and *gxs1-2.3* from *GXS1* and *xut3-2.1* and *xut3-2.2* from *XUT3*) in a hexose transporter null *S. cerevisiae* resulted in substantial increases in growth rate on xylose without affecting growth rates on glucose. They also identified specific amino acid residues (Phe40 of *GXS1* and Glu538 of *XUT1*) where single point mutations can bring about drastic changes in substrate preferences of the transporters. Interestingly, improvement of xylose affinity required the loss of efficiency, namely *V*_*max*_ and *K*_*m*_ simultaneously increased for directed evolution of *C. intermedia GXS1*, while the efficiency of *Sc. stipitis XUT3* could be improved without loosing xylose affinity, suggesting that there might be an inherent limitation in engineering those transporters for optimal performance [122]. Similarly, Reider Apel et al. [123] discovered a novel mutation (Phe79Ser) in *HXT7* that allowed engineered *S. cerevisiae* to display improved xylose uptake and growth on xylose through serial sub-cultures on xylose. Modeling the structure of the mutated Hxt7p predicted that the Phe79Ser mutation is located in a central sugar-binding pore of Hxt7p. The mutant Hxt7p exhibited significantly higher xylose transport efficiency (*V*_*max*_ = 186.4 nmol/min mg) than wild-type Hxt7p (*V*_*max*_ = 101.6 nmol/min mg), whereas the mutated Hxt7p still exhibited a similar level of xylose affinity (*K*_*m*_ = 228.8 mM) to the level of wild-type (*K*_*m*_ = 161.4 mM).

## Production of advanced biofuels and chemicals from xylose by engineered *S. cerevisiae*

Despite numerous metabolic engineering efforts, *S. cerevisiae* still cannot ferment xylose to ethanol as efficiently as glucose. During glucose utilization, *S. cerevisiae* efficiently ferments glucose to ethanol and carbon dioxide by repressing unnecessary metabolic pathways except for alcoholic fermentation regardless of oxygen presence through its peculiar regulatory system, the ‘Crabtree effect’ [124, 125]. On the contrary, xylose utilization by engineered *S. cerevisiae* results in lower metabolic activities of the glycolytic pathway [126–128], and higher expression of genes involved in non-fermentative metabolism through dysregulation of glucose-dependent repression that causes redirection of metabolic fluxes toward byproducts from ethanol production [129–132]. These genetic and metabolic characteristics of engineered *S. cerevisiae* grown on xylose are obstacles to efficient and rapid production of ethanol from xylose. However, at the same time, the metabolic pattern of xylose fermentation might be advantageous for producing advanced biofuels and chemicals as synthetic pathways to produce target molecules can overturn the innate metabolic preference to produce ethanol by *S. cerevisiae*. The following parts introduce recent studies reporting the beneficial effects of xylose utilization on the production of biofuels and chemicals instead of ethanol by engineered *S. cerevisiae*.

### Production of fatty alcohol from xylose

**Fig. 5:**
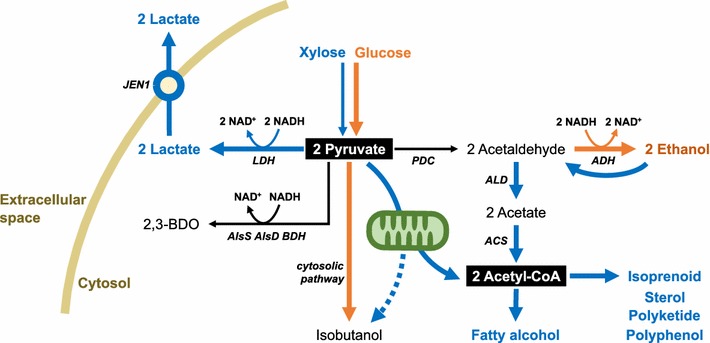
Schematic comparison between glucose (*orange*) and xylose (*blue*) metabolisms in engineered *S. cerevisiae*. *Colored* distinction of *arrows* indicates which sugar induce each metabolic pathway to be predominant. Xylose-derived weaker glycolysis metabolic activity and dysregulation of glucose repressions on cytosolic acetyl-coA synthetic pathway and mitochondrial development (*green*) allowed *S. cerevisiae* to more efficiently produce derivatives of pyruvate and cytosolic acetyl-CoA

1-Hexadecanol, also known as cetyl alcohol and palmityl alcohol, has been used as an emulsifier and a lubricant in various industrial fields, and considered as a potential advanced biofuel. Guo et al. compared productivities of 1-hexadecanol in engineered *S. cerevisiae* under xylose and glucose culture conditions [11]. They previously produced 1-hexadecanol in engineered *S. cerevisiae* by the introduction of fatty acyl-CoA reductase, and further improved its productivity through overexpressing acetyl-CoA carboxylase (*ACC1*), knocking out a negative regulator of phospholipid metabolism, and introducing heterologous cytosolic acetyl-CoA synthetic enzyme, ATP-citrate lyase [133]. The introduction of the optimized XR–XDH–XK expression system and subsequent laboratory evolution on xylose allowed the previous engineered *S. cerevisiae* to produce 1-hexadenanol from xylose. The resulting xylose-utilizing engineered *S. cerevisiae* showed a much higher yield of 1-hexadecanol in both batch (0.10 g/g) and fed-batch fermentation (0.08 g/g) from xylose as compared to glucose (0.03 and <0.01 g/g, respectively) [11, 133]. Three peculiar genetic and physiological characteristics of xylose metabolism are speculated to exhibit the positive effect of xylose on fatty alcohol synthesis in *S. cerevisiae*. First, cytosolic acetyl-CoA, the core intermediate of acyl-CoA synthetic metabolism, could be more efficiently produced under xylose culture conditions compared to glucose, due to dysregulation of glucose-dependent repression on genes related to ethanol re-assimilation (*ADH2*) and cytosolic acetyl-CoA synthesis (*ALD3*, *ALD6*, *ACS1*) [130, 132] (Fig. 5). Second, upregulation of genes encoding enzymes in the tricarboxylic acid cycle or respiratory enzymes in mitochondria [130, 132] probably improved ATP and citrate generation and consequently enhanced cytosolic acetyl-CoA synthesis via endogenous ACS and heterologous ATP citrate lyase (Fig. 5). Lastly, as extracellular xylose cannot sufficiently interact with Snf1p [134], a sensor protein that downregulates the expression of *ACC1* and upregulates β-oxidation [133], xylose culture could prevent inhibition of fatty acid synthesis and degradation of acyl-CoA.

### Production of lactic acid from xylose

Lactic acid (2-hydroxypropanoic acid) is an organic acid with widespread applications in food, cosmetic, pharmaceutical, and polymer industries. Many studies have engineered yeasts with a heterologous lactate dehydrogenase (LDH, EC 1.1.1.27) for biological lactic acid production due to yeast can perform better than lactic acid bacteria under industrial and economic feasible fermentation conditions as recently reviewed [135, 136]. Although furnishing a heterologous LDH allowed engineered *S. cerevisiae* to produce lactic acid, ethanol was still one of the major products under fermentable sugar culture conditions [137–139]. Knockout of PDC and aldehyde dehydrogenase (ADH, EC 1.2.1.3) is a possible solution to reduce metabolic flux toward ethanol, yet the deletion of key metabolic enzymes negatively impact on cell growth [140]. Instead of genetic perturbations for diminishing ethanol production, Turner et al. introduced LDH into an efficient xylose-fermenting *S. cerevisiae*. They demonstrated that the engineered *S. cerevisiae* efficiently produces lactic acid without detectable ethanol accumulation on xylose (Y_lactate/xylose_ = 0.69 g/g xylose, Y_ethanol/xylose_ < 0.01 g/g xylose), while the engineered yeast produced lactic acid and ethanol simultaneously at 2:3 ratios on glucose (Y_lactate/xylose_ = 0.22 g/g glucose, Y_ethanol/xylose_ = 0.31 g/g glucose) [12]. Weaker metabolic activity of glycolysis on xylose [126–128] is a probable interpretation of this phenomenon. LDH competes with PDC for pyruvate which is the final product of glycolysis as well as the branch point metabolite between lactate and ethanol pathways. As LDH has smaller K_M_ value than PDC [12], the inefficient glycolytic metabolism under xylose culture conditions would allow LDH to direct more metabolic flux from pyruvate than endogenous PDC. Additionally, the expression level of *JEN1*, a lactate-proton symporter coding gene, is upregulated under xylose through dysregulation of glucose-dependent repression [130]. Efficient transporting lactic acid to the outside of yeast cells via Jen1p increases productivity and yield of lactic acid as LDH reaction is reversible and allosterically inhibited by lactate [137] (Fig. 5).

### Perspectives and remaining challenges

Cellulosic ethanol is being commercialized, but production of more functional and valuable molecules than ethanol from cellulosic sugars is also anticipated. Acetyl-CoA is an important precursor for many valuable biochemical products. However, there are hurdles impeding ample biosynthesis of acetyl-CoA-derived products in *S. cerevisiae*. First, acetyl-CoA synthesis in *S. cerevisiae* is compartmentalized in four different cellular spaces: mitochondria, cytosol, peroxisome, and nucleus. While many valuable acetyl-CoA derived chemicals, such as isoprenoids, sterols, polyketides, polyphenols, and waxes, are synthesized in the cytosol, cytosolic acetyl-CoA synthesis in yeast is limited. Second, the cytosolic acetyl-CoA synthetic pathway shares acetaldehyde as an intermediate but acetaldehyde is drained by the ethanol producing pathway. Third, the native ACS reaction of *S. cerevisiae* is energetically expensive [141]. As distinctive features of *S. cerevisiae* xylose metabolism mitigate the three hurdles, we can speculate that the positive effect of xylose utilization on the fatty alcohol production would be extended to the biosynthesis of other value-added molecules deriving from cytosolic acetyl-CoA (Fig. 5). In-depth studies about the PK-ACK/PTA pathway (Fig. 1)—a carbon conserving cytosolic acetyl-CoA synthetic metabolism—would be necessary to further enhance the yield of cytosolic acetyl-CoA derivatives under xylose conditions.

In the same manner, engineered *S. cerevisiae* would exhibit a higher yield of not only lactic acid but also other molecule derived from pyruvate or other glycolysis intermediates. However, prior studies reported contrary results to this expectation. No meaningful improvements in the production of 2,3-butanediol [142] and isobutanol [143] which are derived from pyruvate have been reported under xylose conditions. A notable difference between the enhanced lactic acid production and the latter ineffectual production of 2,3-butanediol and isobutanol is the order of metabolic engineering efforts. Namely, for the production of 2,3-butanediol and isobutanol, xylose pathways were introduced after optimizing the production pathway from glucose, while the lactic acid study furnished LDH into a host strain which had already optimized for xylose fermentation.

To produce 2,3-butanediol from xylose by engineered yeast, the XR–XDH pathway was introduced to a pyruvate decarboxylase deficient (*Δpdc1*, *Δpdc5*) *S. cerevisiae* harboring a mutation in *MTH1* (A81P) and a heterologous 2,3-butanediol synthetic pathway [142, 144]. The 2,3-butanediol synthetic pathway consisting of AlsS (acetolactate synthase, EC 2.2.1.6), AlsD (acetolactate decarboxylase, EC 4.1.1.5), and BDH (2,3-butanediol dehydrogenase, EC 1.1.1.4) regenerates a half amount of NAD^+^ regenerated from the LDH reaction which can regenerate an equivalent amount of NAD^+^ as compared to ADH reaction. This insufficient NAD^+^ regeneration from 2,3-butanediol production and surplus NADH derived from the XR–XDH pathway induce NADH oxidation via glycerol synthesis during xylose conversion to 2,3-butanediol and consequently resulted in lower 2,3-butanediol yields compared to that from glucose (Fig. 5). Considering redox imbalances of the XR–XDH pathway and 2,3-butanediol pathway, introduction of the XI pathway, which does not require cofactors, would be more appropriate approach to observe the positive xylose effect on 2,3-butanetiol production.

Similarly, the isobutanol study also introduced the XI pathway into an existing *S. cerevisiae* engineered for efficient isobutanol production from glucose through re-localization of mitochondrial Ilv2p (acetolactate synthase), Ilv5p (keto-acid reductoisomerase, EC 1.1.1.86), and Ilv3p (dihydroxyacid dehydratase, EC 4.2.1.9) to cytosol [145] (Fig. 5). Although the engineered *S. cerevisiae* successfully overcame the loss of mitochondrial function and increased isobutanol production on glucose, the re-localization of mitochondrial pathway was not an appropriate strategy on xylose as utilization of non-fermentable sugars dysregulates glucose-dependent repression on the development of mitochondria and induces its metabolism [146, 147]. In that respect, we can assume that reverse localization of cytosolic pathway into mitochondria [148] is a proper strategy to improve isobutanol production from xylose (Fig. 5).

Inadequate optimization of xylose pathways could also be a considerable cause of the inefficient production of the 2,3-butanediol and isobutanol from xylose as compared to glucose. Especially, the *MTH1* mutation (A81P) alleviated the toxic effects on cell growth by PDC knockout via decreased sugar influx during glucose fermentation [144], yet higher *MTH1* expression levels under xylose conditions [130] could additionally reduce xylose influx and further decrease 2,3-butanediol productivity as compared to glucose.

We can come to a conclusion that xylose might serve better than glucose as a substrate for producing advanced biofuels and chemicals derived from cytosolic acetyl-CoA, pyruvate, and other intermediates which must compete with rigid overflow metabolism to be acquired in *S. cerevisiae.* Xylose utilization might lead to undesirable redox and energy balances [126]. Nonetheless, xylose is an effective sugar to overcome the Crabtree effect and to redirect *S. cerevisiae* metabolism for better production of above target biofuels and chemicals instead of producing ethanol [130–132, 149]. To maixmize the benficial effects of xylose, optimization of the xylose metabolic pathway is pre-requisite for efficient conversion of xylose to target molecules. In addition, culture conditions, synthetic pathways of target molecules, and additional optimization on endogenous metabolisms will need to be carefully conisered in the context of xylose metabolism.

## Concluding remarks

As xylose is the second most abundant monosaccharide in lignocellulosic materials, many studies have searched and developed efficient xylose utilizing microorganisms for economic and sustainable processes capable of converting lignocellulosic biomass to fuels and chemicals. Instead of using natural xylose-fermenting yeast strains, *S. cerevisiae* has been thoroughly engineered to assimilate xylose due to its robustness under industrial fermentation conditions. The XR–XDH pathway and the XI pathway are being used to develop efficient xylose-fermenting *S. cerevisiae*. The major drawback—dual cofactor preference—of the XR–XDH pathway, was minimized by protein engineering, controlling activity ratio of XR/XDH, and adding heterologous electron sink reactions. Conversely, the cofactor imbalance also could act as a driving force to utilize acetate in the hydrolysate and carbon dioxide for increasing ethanol yield via novel synthetic pathways that require electron acceptors for the oxidation of NADH. Insufficient expression and inferior kinetic properties of XI were overcome through codon optimization, increasing gene dosage, and directed protein evolution. Additionally, xylose assimilation via XI could be further enhanced through perturbations on endogenous iron-sulfur biosynthesis and regulation mechanism for glycolytic fluxes. Xylose would serve as a better sugar than glucose to produce advanced biofuels and chemicals whose synthetic pathway was regulated by glucose repression. In particular, 1-hexadecanol and lactic acid, derived from cytosolic acetyl-CoA and pyruvate, could be more efficiently synthesized through xylose utilization under appropriate metabolic engineering approaches considering xylose metabolism of *S. cerevisiae*. Initial motivation of xylose utilization by engineered yeast was because xylose was an abundant sugar in cellulosic hydrolysates. However, more applications to exploit the unique metabolic regulation by xylose can be considered in the future.

## Abbreviations

### Enzymes

XR: xylose reductase; XDH: xylitol dehydrogenase; XI: xylose isomerase; XK: xylulokinase; PK: phosphoketolase; ACK: acetate kinase; PTA: phosphotransacetylase; ACS: acetyl-CoA synthase; AADH: acetylating acetaldehyde dehydrogenase; RuBisCO: ribulose-1,5-bisphosphate carboxylase/oxygenase; PRK: phosphoribulokinase; LDH: lactate dehydrogenase; PDC: pyruvate decarboxylase; ADH: aldehyde dehydrogenase; AlsS: acetolactate synthase; AlsD: acetolactate decarboxylase; BDH: 2,3-butanediol dehydrogenase

### Metabolites

ATP: adenosine triphosphate; cAMP: cyclic adenosine monophosphate; 6-PGL: 6-phosphoglucono-1,5-lactone; 6-PG: 6-phosphogluconate; GA-3-P: glyceraldehyde-3-phosphate

## References

[1] Mosier N, Wyman C, Dale B, Elander R, Lee YY, Holtzapple M (2005). Features of promising technologies for pretreatment of lignocellulosic biomass. Bioresour Technol.

[2] Kim SR, Ha S-J, Wei N, Oh EJ, Jin Y-S (2012). Simultaneous co-fermentation of mixed sugars: a promising strategy for producing cellulosic ethanol. Trends Biotechnol.

[3] Auesukaree C, Damnernsawad A, Kruatrachue M, Pokethitiyook P, Boonchird C, Kaneko Y (2009). Genome-wide identification of genes involved in tolerance to various environmental stresses in *Saccharomyces cerevisiae*. J Appl Genet..

[4] Hong K-K, Nielsen J (2012). Metabolic engineering of *Saccharomyces cerevisiae*: a key cell factory platform for future biorefineries. Cell Mol Life Sci CMLS.

[5] Knoshaug EP, Zhang M (2009). Butanol tolerance in a selection of microorganisms. Appl Biochem Biotechnol.

[6] Kim SR, Park Y-C, Jin Y-S, Seo J-H (2013). Strain engineering of *Saccharomyces cerevisiae* for enhanced xylose metabolism. Biotechnol Adv.

[7] Young E, Lee S-M, Alper H (2010). Optimizing pentose utilization in yeast: the need for novel tools and approaches. Biotechnol Biofuels.

[8] Zhang G-C, Liu J-J, Kong II, Kwak S, Jin Y-S (2015). Combining C6 and C5 sugar metabolism for enhancing microbial bioconversion. Curr Opin Chem Biol.

[9] Sànchez Nogué V, Karhumaa K (2015). Xylose fermentation as a challenge for commercialization of lignocellulosic fuels and chemicals. Biotechnol Lett.

[10] Harner NK, Wen X, Bajwa PK, Austin GD, Ho C-Y, Habash MB (2015). Genetic improvement of native xylose-fermenting yeasts for ethanol production. J Ind Microbiol Biotechnol.

[11] Guo W, Sheng J, Zhao H, Feng X (2016). Metabolic engineering of *Saccharomyces cerevisiae* to produce 1-hexadecanol from xylose. Microb Cell Factories.

[12] Turner TL, Zhang G-C, Kim SR, Subramaniam V, Steffen D, Skory CD (2015). Lactic acid production from xylose by engineered *Saccharomyces cerevisiae* without *PDC* or *ADH* deletion. Appl Microbiol Biotechnol.

[13] Bhosale SH, Rao MB, Deshpande VV (1996). Molecular and industrial aspects of glucose isomerase. Microbiol Rev.

[14] Harhangi HR, Akhmanova AS, Emmens R, van der Drift C, de Laat WTAM, van Dijken JP (2003). Xylose metabolism in the anaerobic fungus *Piromyces* sp. strain E2 follows the bacterial pathway. Arch Microbiol.

[15] Madhavan A, Tamalampudi S, Ushida K, Kanai D, Katahira S, Srivastava A (2009). Xylose isomerase from polycentric fungus *Orpinomyces*: gene sequencing, cloning, and expression in *Saccharomyces cerevisiae* for bioconversion of xylose to ethanol. Appl Microbiol Biotechnol.

[16] Stincone A, Prigione A, Cramer T, Wamelink MMC, Campbell K, Cheung E (2015). The return of metabolism: biochemistry and physiology of the pentose phosphate pathway. Biol Rev Camb Philos Soc.

[17] Grant CM, Collinson LP, Roe JH, Dawes IW (1996). Yeast glutathione reductase is required for protection against oxidative stress and is a target gene for yAP-1 transcriptional regulation. Mol Microbiol.

[18] Whitworth DA, Ratledge C (1977). Phosphoketolase in *Rhodotorula graminis* and Other yeasts. Microbiology.

[19] Sánchez B, Zúñiga M, González-Candelas F, de los Reyes-Gavilán CG, Margolles A (2010). Bacterial and eukaryotic phosphoketolases: phylogeny, distribution and evolution. J Mol Microbiol Biotechnol..

[20] Ingram-Smith C, Martin SR, Smith KS (2006). Acetate kinase: not just a bacterial enzyme. Trends Microbiol.

[21] Meadows AL, Hawkins KM, Tsegaye Y, Antipov E, Kim Y, Raetz L (2016). Rewriting yeast central carbon metabolism for industrial isoprenoid production. Nature.

[22] Jeffries TW (1983). Utilization of xylose by bacteria, yeasts, and fungi. Adv Biochem Eng Biotechnol.

[23] du Preez JC, Prior BA (1985). A quantitative screening of some xylose-fermenting yeast isolates. Biotechnol Lett.

[24] Riley R, Haridas S, Wolfe KH, Lopes MR, Hittinger CT, Göker M (2016). Comparative genomics of biotechnologically important yeasts. Proc Natl Acad Sci USA..

[25] Jeffries TW, Van Vleet JRH (2009). *Pichia stipitis* genomics, transcriptomics, and gene clusters. FEMS Yeast Res.

[26] Nguyen NH, Suh S-O, Marshall CJ, Blackwell M (2006). Morphological and ecological similarities: wood-boring beetles associated with novel xylose-fermenting yeasts, *Spathaspora passalidarum* gen. sp. nov. and *Candida jeffriesii* sp. nov. Mycol Res.

[27] Hou X (2012). Anaerobic xylose fermentation by *Spathaspora passalidarum*. Appl Microbiol Biotechnol.

[28] Wohlbach DJ, Kuo A, Sato TK, Potts KM, Salamov AA, Labutti KM (2011). Comparative genomics of xylose-fermenting fungi for enhanced biofuel production. Proc Natl Acad Sci USA.

[29] Laplace JM, Delgenes JP, Moletta R, Navarro JM (1991). Combined alcoholic fermentation of d-xylose and d-glucose by four selected microbial strains: process considerations in relation to ethanol tolerance. Biotechnol Lett.

[30] Hou X, Yao S (2012). Improved inhibitor tolerance in xylose-fermenting yeast *Spathaspora passalidarum* by mutagenesis and protoplast fusion. Appl Microbiol Biotechnol.

[31] du Preez JC, van Driessel B, Prior BA (1989). D-xylose fermentation by *Candida shehatae* and *pichia stipitis* at low dissolved oxygen levels in fed-batch cultures. Biotechnol Lett.

[32] Goffeau A, Barrell BG, Bussey H, Davis RW, Dujon B, Feldmann H (1996). Life with 6000 genes. Science.

[33] Richard P, Toivari MH, Penttilä M (2000). The role of xylulokinase in *Saccharomyces cerevisiae* xylulose catabolism. FEMS Microbiol Lett.

[34] Amore R, Wilhelm M, Hollenberg CP (1989). The fermentation of xylose—an analysis of the expression of *Bacillus* and *Actinoplanes* xylose isomerase genes in yeast. Appl Microbiol Biotechnol.

[35] Moes CJ, Pretorius IS, van Zyl WH (1996). Cloning and expression of the *Clostridium thermosulfurogenes* D-xylose isomerase gene (*xylA*) in *Saccharomyces cerevisiae*. Biotechnol Lett.

[36] Sarthy AV, McConaughy BL, Lobo Z, Sundstrom JA, Furlong CE, Hall BD (1987). Expression of the *Escherichia coli* xylose isomerase gene in *Saccharomyces cerevisiae*. Appl Environ Microbiol.

[37] Parachin NS, Gorwa-Grauslund MF (2011). Isolation of xylose isomerases by sequence- and function-based screening from a soil metagenomic library. Biotechnol Biofuels.

[38] Kuyper M, Hartog MMP, Toirkens MJ, Almering MJH, Winkler AA, van Dijken JP (2005). Metabolic engineering of a xylose-isomerase-expressing *Saccharomyces cerevisiae* strain for rapid anaerobic xylose fermentation. FEMS Yeast Res.

[39] Kuyper M, Harhangi HR, Stave AK, Winkler AA, Jetten MSM, de Laat WTAM (2003). High-level functional expression of a fungal xylose isomerase: the key to efficient ethanolic fermentation of xylose by *Saccharomyces cerevisiae*?. FEMS Yeast Res.

[40] van Maris AJA, Winkler AA, Kuyper M, de Laat WTAM, van Dijken JP, Pronk JT (2007). Development of efficient xylose fermentation in *Saccharomyces cerevisiae*: xylose isomerase as a key component. Adv Biochem Eng Biotechnol.

[41] Walfridsson M, Bao X, Anderlund M, Lilius G, Bülow L, Hahn-Hägerdal B (1996). Ethanolic fermentation of xylose with *Saccharomyces cerevisiae* harboring the *Thermus thermophilus xylA* gene, which expresses an active xylose (glucose) isomerase. Appl Environ Microbiol.

[42] Brat D, Boles E, Wiedemann B (2009). Functional expression of a bacterial xylose isomerase in *Saccharomyces cerevisiae*. Appl Environ Microbiol.

[43] Ha SJ, Kim SR, Choi JH, Park MS, Jin Y-S (2011). Xylitol does not inhibit xylose fermentation by engineered *Saccharomyces cerevisiae* expressing *xylA* as severely as it inhibits xylose isomerase reaction in vitro. Appl Microbiol Biotechnol.

[44] Zhou H, Cheng J-S, Wang BL, Fink GR, Stephanopoulos G (2012). Xylose isomerase overexpression along with engineering of the pentose phosphate pathway and evolutionary engineering enable rapid xylose utilization and ethanol production by *Saccharomyces cerevisiae*. Metab Eng.

[45] Wiedemann B, Boles E (2008). Codon-optimized bacterial genes improve L-arabinose fermentation in recombinant *Saccharomyces cerevisiae*. Appl Environ Microbiol.

[46] Dos Santos LV, Carazzolle MF, Nagamatsu ST, Sampaio NMV, Almeida LD, Pirolla RAS (2016). Unraveling the genetic basis of xylose consumption in engineered *Saccharomyces cerevisiae* strains. Sci Rep.

[47] Lee S-M, Jellison T, Alper HS (2012). Directed evolution of xylose isomerase for improved xylose catabolism and fermentation in the yeast *Saccharomyces cerevisiae*. Appl Environ Microbiol.

[48] Sato TK, Tremaine M, Parreiras LS, Hebert AS, Myers KS, Higbee AJ (2016). Directed evolution reveals unexpected epistatic interactions that alter metabolic regulation and enable anaerobic xylose use by *Saccharomyces cerevisiae*. PLoS Genet.

[49] Ariño J, Ramos J, Sychrová H (2010). Alkali metal cation transport and homeostasis in yeasts. Microbiol Mol Biol Rev MMBR..

[50] Garland SA, Hoff K, Vickery LE, Culotta VC (1999). *Saccharomyces cerevisiae ISU1* and *ISU2*: members of a well-conserved gene family for iron-sulfur cluster assembly. J Mol Biol.

[51] Kovalevsky AY, Hanson L, Fisher SZ, Mustyakimov M, Mason SA, Forsyth VT (1993). Metal ion roles and the movement of hydrogen during reaction catalyzed by D-xylose isomerase: a joint x-ray and neutron diffraction study. Struct Lond Engl.

[52] Garay-Arroyo A, Covarrubias AA (1999). Three genes whose expression is induced by stress in *Saccharomyces cerevisiae*. Yeast Chichester Engl..

[53] Tanaka K, Nakafuku M, Satoh T, Marshall MS, Gibbs JB, Matsumoto K (1990). *S. cerevisiae* genes *IRA1* and *IRA2* encode proteins that may be functionally equivalent to mammalian ras GTPase activating protein. Cell.

[54] Santangelo GM (2006). Glucose signaling in *Saccharomyces cerevisiae*. Microbiol Mol Biol Rev MMBR..

[55] Ho NW, Chen Z, Brainard AP (1998). Genetically engineered *Saccharomyces yeast* capable of effective cofermentation of glucose and xylose. Appl Environ Microbiol.

[56] Jin Y-S, Lee TH, Choi YD, Ryu YW, Seo JH (2000). Conversion of xylose to ethanol by recombinant *Saccharomyces cerevisiae* containing genes for xylose reductase and xylitol dehydrogenase from *Pichia stipitis*. J Microbiol Biotechnol.

[57] Kim SR, Skerker JM, Kang W, Lesmana A, Wei N, Arkin AP (2013). Rational and evolutionary engineering approaches uncover a small set of genetic changes efficient for rapid xylose fermentation in *Saccharomyces cerevisiae*. PLoS ONE.

[58] Kötter P, Ciriacy M (1993). Xylose fermentation by *Saccharomyces cerevisiae*. Appl Microbiol Biotechnol.

[59] Walfridsson M, Hallborn J, Penttilä M, Keränen S, Hahn-Hägerdal B (1995). Xylose-metabolizing *Saccharomyces cerevisiae* strains overexpressing the *TKL1* and *TAL1* genes encoding the pentose phosphate pathway enzymes transketolase and transaldolase. Appl Environ Microbiol.

[60] Karhumaa K, Garcia Sanchez R, Hahn-Hägerdal B, Gorwa-Grauslund MF (2007). Comparison of the xylose reductase-xylitol dehydrogenase and the xylose isomerase pathways for xylose fermentation by recombinant *Saccharomyces cerevisiae*. Microb Cell Factories.

[61] Li X, Park A, Estrela R, Kim SR, Jin YS, Cate JHD (2016). Comparison of xylose fermentation by two high-performance engineered strains of *Saccharomyces cerevisiae*. Biotechnol Rep..

[62] Ghosh A, Zhao H, Price ND (2011). Genome-scale consequences of cofactor balancing in engineered pentose utilization pathways in *Saccharomyces cerevisiae*. PLoS ONE.

[63] Khoury GA, Fazelinia H, Chin JW, Pantazes RJ, Cirino PC, Maranas CD (2009). Computational design of *Candida boidinii* xylose reductase for altered cofactor specificity. Prot Sci Publ Prot Soc..

[64] Leitgeb S, Petschacher B, Wilson DK, Nidetzky B (2005). Fine tuning of coenzyme specificity in family 2 aldo-keto reductases revealed by crystal structures of the Lys-274–>Arg mutant of *Candida tenuis* xylose reductase (AKR2B5) bound to NAD^+^ and NADP^+^. FEBS Lett.

[65] Liang L, Zhang J, Lin Z (2007). Altering coenzyme specificity of *Pichia stipitis* xylose reductase by the semi-rational approach CASTing. Microb Cell Factories..

[66] Petschacher B, Leitgeb S, Kavanagh KL, Wilson DK, Nidetzky B (2005). The coenzyme specificity of *Candida tenuis* xylose reductase (AKR2B5) explored by site-directed mutagenesis and X-ray crystallography. Biochem J.

[67] Ehrensberger AH, Elling RA, Wilson DK (1993). Structure-guided engineering of xylitol dehydrogenase cosubstrate specificity. Struct Lond Engl.

[68] Watanabe S, Kodaki T, Makino K (2005). Complete reversal of coenzyme specificity of xylitol dehydrogenase and increase of thermostability by the introduction of structural zinc. J Biol Chem.

[69] Bengtsson O, Hahn-Hägerdal B, Gorwa-Grauslund MF (2009). Xylose reductase from *Pichia stipitis* with altered coenzyme preference improves ethanolic xylose fermentation by recombinant *Saccharomyces cerevisiae*. Biotechnol Biofuels.

[70] Lee S-H, Kodaki T, Park Y-C, Seo J-H (2012). Effects of NADH-preferring xylose reductase expression on ethanol production from xylose in xylose-metabolizing recombinant *Saccharomyces cerevisiae*. J Biotechnol.

[71] Watanabe S, Abu Saleh A, Pack SP, Annaluru N, Kodaki T, Makino K (2007). Ethanol production from xylose by recombinant *Saccharomyces cerevisiae* expressing protein-engineered NADH-preferring xylose reductase from *Pichia stipitis*. Microbiol Read Engl.

[72] Matsushika A, Watanabe S, Kodaki T, Makino K, Inoue H, Murakami K (2008). Expression of protein engineered NADP+-dependent xylitol dehydrogenase increases ethanol production from xylose in recombinant *Saccharomyces cerevisiae*. Appl Microbiol Biotechnol.

[73] Watanabe S, Saleh AA, Pack SP, Annaluru N, Kodaki T, Makino K (2007). Ethanol production from xylose by recombinant *Saccharomyces cerevisiae* expressing protein engineered NADP^+^-dependent xylitol dehydrogenase. J Biotechnol.

[74] Cadete RM, de Las Heras AM, Sandström AG, Ferreira C, Gírio F, Gorwa-Grauslund M-F (2016). Exploring xylose metabolism in *Spathaspora species*: *XYL1.2* from *Spathaspora passalidarum* as the key for efficient anaerobic xylose fermentation in metabolic engineered *Saccharomyces cerevisiae*. Biotechnol Biofuels.

[75] Eliasson A (2001). Hofmeyr J-HS, Pedler S, Hahn-Hägerdal B. The xylose reductase/xylitol dehydrogenase/xylulokinase ratio affects product formation in recombinant xylose-utilising *Saccharomyces cerevisiae*. Enzyme Microb Technol.

[76] Walfridsson M, Anderlund M, Bao X, Hahn-Hägerdal B (1997). Expression of different levels of enzymes from the *Pichia stipitis XYL1* and *XYL2* genes in *Saccharomyces cerevisiae* and its effects on product formation during xylose utilisation. Appl Microbiol Biotechnol.

[77] Jin Y-S, Jeffries TW (2003). Changing flux of xylose metabolites by altering expression of xylose reductase and xylitol dehydrogenase in recombinant *Saccharomyces cerevisiae*. Appl Biochem Biotechnol.

[78] Kim SR, Kwee NR, Kim H, Jin Y-S (2013). Feasibility of xylose fermentation by engineered *Saccharomyces cerevisiae* overexpressing endogenous aldose reductase (*GRE3*), xylitol dehydrogenase (*XYL2*), and xylulokinase (*XYL3*) from *Scheffersomyces stipitis*. FEMS Yeast Res.

[79] Kim SR, Ha SJ, Kong II, Jin YS (2012). High expression of *XYL2* coding for xylitol dehydrogenase is necessary for efficient xylose fermentation by engineered *Saccharomyces cerevisiae*. Metab Eng.

[80] Jeppsson M, Johansson B, Hahn-Hägerdal B, Gorwa-Grauslund MF (2002). Reduced oxidative pentose phosphate pathway flux in recombinant xylose-utilizing *Saccharomyces cerevisiae* strains improves the ethanol yield from xylose. Appl Environ Microbiol.

[81] Verho R, Londesborough J, Penttilä M, Richard P (2003). Engineering redox cofactor regeneration for improved pentose fermentation in *Saccharomyces cerevisiae*. Appl Environ Microbiol.

[82] Roca C, Nielsen J, Olsson L (2003). Metabolic engineering of ammonium assimilation in xylose-fermenting *Saccharomyces cerevisiae* improves ethanol production. Appl Environ Microbiol.

[83] Zhang GC, Liu JJ, Ding WT (2012). Decreased xylitol formation during xylose fermentation in *Saccharomyces cerevisiae* due to overexpression of water-forming NADH oxidase. Appl Environ Microbiol.

[84] Wei N, Quarterman J, Kim SR, Cate JHD, Jin YS (2013). Enhanced biofuel production through coupled acetic acid and xylose consumption by engineered yeast. Nat Commun.

[85] Palmqvist E, Hahn-Hägerdal B (2000). Fermentation of lignocellulosic hydrolysates. II: inhibitors and mechanisms of inhibition. Bioresour Technol.

[86] Zhang GC, Kong II, Wei N, Peng D, Turner TL, Sung BH (2016). Optimization of an acetate reduction pathway for producing cellulosic ethanol by engineered yeast. Biotechnol Bioeng..

[87] Xia PF, Zhang GC, Walker B, Seo SO, Kwak S, Liu JJ (2017). Recycling carbon dioxide during xylose fermentation by engineered *Saccharomyces cerevisiae*. ACS Synth Biol..

[88] Guadalupe-Medina V, Wisselink HW, Luttik MA, de Hulster E, Daran JM, Pronk JT (2013). Carbon dioxide fixation by Calvin-cycle enzymes improves ethanol yield in yeast. Biotechnol Biofuels.

[89] Li YJ, Wang MM, Chen YW, Wang M, Fan LH, Tan TW (2017). Engineered yeast with a CO2-fixation pathway to improve the bio-ethanol production from xylose-mixed sugars. Sci Rep..

[90] Jin YS, Alper H, Yang YT, Stephanopoulos G (2005). Improvement of xylose uptake and ethanol production in recombinant *Saccharomyces cerevisiae* through an inverse metabolic engineering approach. Appl Environ Microbiol.

[91] Johansson B, Christensson C, Hobley T, Hahn-Hägerdal B (2001). Xylulokinase overexpression in two strains of *Saccharomyces cerevisiae* also expressing xylose reductase and xylitol dehydrogenase and its effect on fermentation of xylose and lignocellulosic hydrolysate. Appl Environ Microbiol.

[92] Lee TH, Kim MD, Park YC, Bae SM, Ryu YW, Seo JH (2003). Effects of xylulokinase activity on ethanol production from D-xylulose by recombinant *Saccharomyces cerevisiae*. J Appl Microbiol.

[93] Matsushika A, Sawayama S (2008). Efficient bioethanol production from xylose by recombinant *Saccharomyces cerevisiae* requires high activity of xylose reductase and moderate xylulokinase activity. J Biosci Bioeng.

[94] Toivari MH, Aristidou A, Ruohonen L, Penttilä M (2001). Conversion of xylose to ethanol by recombinant *Saccharomyces cerevisiae*: importance of xylulokinase (*XKS1*) and oxygen availability. Metab Eng.

[95] Lee S-M, Jellison T, Alper HS (2014). Systematic and evolutionary engineering of a xylose isomerase-based pathway in *Saccharomyces cerevisiae* for efficient conversion yields. Biotechnol Biofuels.

[96] Träff KL, Otero Cordero RR, van Zyl WH, Hahn-Hägerdal B (2001). Deletion of the *GRE3* aldose reductase gene and its influence on xylose metabolism in recombinant strains of *Saccharomyces cerevisiae* expressing the *xylA* and *XKS1* genes. Appl Environ Microbiol.

[97] Usher J, Balderas-Hernandez V, Quon P, Gold ND, Martin VJJ, Mahadevan R (2011). Chemical and synthetic genetic array analysis identifies genes that suppress xylose utilization and fermentation in *Saccharomyces cerevisiae*. G3 Bethesda Md.

[98] Jin YS, Ni H, Laplaza JM, Jeffries TW (2003). Optimal growth and ethanol production from xylose by recombinant *Saccharomyces cerevisiae* require moderate D-xylulokinase activity. Appl Environ Microbiol.

[99] Jin Y-S, Jones S, Shi NQ, Jeffries TW (2002). Molecular cloning of *XYL3* (D-xylulokinase) from *Pichia stipitis* and characterization of its physiological function. Appl Environ Microbiol.

[100] Deng XX, Ho NW (1990). Xylulokinase activity in various yeasts including *Saccharomyces cerevisiae* containing the cloned xylulokinase gene. Appl Biochem Biotechnol..

[101] Rodriguez-Peña JM, Cid VJ, Arroyo J, Nombela C (1998). The YGR194c (*XKS1*) gene encodes the xylulokinase from the budding yeast *Saccharomyces cerevisiae*. FEMS Microbiol Lett.

[102] Ni H, Laplaza JM, Jeffries TW (2007). Transposon mutagenesis to improve the growth of recombinant *Saccharomyces cerevisiae* on D-xylose. Appl Environ Microbiol.

[103] Johansson B, Hahn-Hägerdal B (2002). The non-oxidative pentose phosphate pathway controls the fermentation rate of xylulose but not of xylose in *Saccharomyces cerevisiae* TMB3001. FEMS Yeast Res.

[104] Bera AK, Ho NWY, Khan A, Sedlak M (2011). A genetic overhaul of *Saccharomyces cerevisiae* 424A(LNH-ST) to improve xylose fermentation. J Ind Microbiol Biotechnol.

[105] Matsushika A, Goshima T, Fujii T, Inoue H, Sawayama S, Yano S (2012). Characterization of non-oxidative transaldolase and transketolase enzymes in the pentose phosphate pathway with regard to xylose utilization by recombinant *Saccharomyces cerevisiae*. Enzyme Microb Technol.

[106] Xu H, Kim S, Sorek H, Lee Y, Jeong D, Kim J (2016). *PHO13* deletion-induced transcriptional activation prevents sedoheptulose accumulation during xylose metabolism in engineered *Saccharomyces cerevisiae*. Metab Eng.

[107] Karhumaa K, Hahn-Hägerdal B, Gorwa-Grauslund M-F (2005). Investigation of limiting metabolic steps in the utilization of xylose by recombinant *Saccharomyces cerevisiae* using metabolic engineering. Yeast.

[108] Fujitomi K, Sanda T, Hasunuma T, Kondo A (2012). Deletion of the *PHO13* gene in *Saccharomyces cerevisiae* improves ethanol production from lignocellulosic hydrolysate in the presence of acetic and formic acids, and furfural. Bioresour Technol.

[109] Van Vleet JH, Jeffries TW, Olsson L (2008). Deleting the para-nitrophenyl phosphatase (pNPPase), *PHO13*, in recombinant *Saccharomyces cerevisiae* improves growth and ethanol production on D-xylose. Metab Eng.

[110] Kim SR, Xu H, Lesmana A, Kuzmanovic U, Au M, Florencia C (2015). Deletion of *PHO13*, encoding haloacid dehalogenase type IIA phosphatase, results in upregulation of the pentose phosphate pathway in *Saccharomyces cerevisiae*. Appl Environ Microbiol.

[111] de Jong BW, Shi S, Siewers V, Nielsen J (2014). Improved production of fatty acid ethyl esters in *Saccharomyces cerevisiae* through up-regulation of the ethanol degradation pathway and expression of the heterologous phosphoketolase pathway. Microb Cell Factories.

[112] Kocharin K, Siewers V, Nielsen J (2013). Improved polyhydroxybutyrate production by *Saccharomyces cerevisiae* through the use of the phosphoketolase pathway. Biotechnol Bioeng.

[113] Sonderegger M, Schümperli M, Sauer U (2004). Metabolic engineering of a phosphoketolase pathway for pentose catabolism in *Saccharomyces cerevisiae*. Appl Environ Microbiol.

[114] Sedlak M, Ho NWY (2004). Characterization of the effectiveness of hexose transporters for transporting xylose during glucose and xylose co-fermentation by a recombinant *Saccharomyces* yeast. Yeast Chichester Engl.

[115] Hamacher T, Becker J, Gárdonyi M, Hahn-Hägerdal B, Boles E (2002). Characterization of the xylose-transporting properties of yeast hexose transporters and their influence on xylose utilization. Microbiology.

[116] Saloheimo A, Rauta J, Stasyk OV, Sibirny AA, Penttilä M, Ruohonen L (2007). Xylose transport studies with xylose-utilizing *Saccharomyces cerevisiae* strains expressing heterologous and homologous permeases. Appl Microbiol Biotechnol.

[117] Kotyk A (1965). Properties of the sugar carrier in baker’s yeast. Folia Microbiol (Praha).

[118] Jojima T, Omumasaba CA, Inui M, Yukawa H (2010). Sugar transporters in efficient utilization of mixed sugar substrates: current knowledge and outlook. Appl Microbiol Biotechnol.

[119] Young E, Poucher A, Comer A, Bailey A, Alper H (2011). Functional survey for heterologous sugar transport proteins, using *Saccharomyces cerevisiae* as a host. Appl Environ Microbiol.

[120] Runquist D, Fonseca C, Rådström P, Spencer-Martins I, Hahn-Hägerdal B (2009). Expression of the Gxf1 transporter from *Candida intermedia* improves fermentation performance in recombinant xylose-utilizing *Saccharomyces cerevisiae*. Appl Microbiol Biotechnol.

[121] Moon J, Lewis Liu Z, Ma M, Slininger PJ (2013). New genotypes of industrial yeast *Saccharomyces cerevisiae* engineered with YXI and heterologous xylose transporters improve xylose utilization and ethanol production. Biocatal Agric Biotechnol.

[122] Young EM, Comer AD, Huang H, Alper HS (2012). A molecular transporter engineering approach to improving xylose catabolism in *Saccharomyces cerevisiae*. Metab Eng.

[123] Reider Apel A, Ouellet M, Szmidt-Middleton H, Keasling JD, Mukhopadhyay A (2016). Evolved hexose transporter enhances xylose uptake and glucose/xylose co-utilization in *Saccharomyces cerevisiae*. Sci Rep..

[124] Pfeiffer T, Morley A (2014). An evolutionary perspective on the Crabtree effect. Front Mol Biosci.

[125] De Deken RH (1966). The Crabtree effect: a regulatory system in yeast. J Gen Microbiol.

[126] Matsushika A, Nagashima A, Goshima T, Hoshino T (2013). Fermentation of xylose causes inefficient metabolic state due to carbon/energy starvation and reduced glycolytic flux in recombinant industrial *Saccharomyces cerevisiae*. PLoS ONE.

[127] Pitkänen J-P, Aristidou A, Salusjärvi L, Ruohonen L, Penttilä M (2003). Metabolic flux analysis of xylose metabolism in recombinant *Saccharomyces cerevisiae* using continuous culture. Metab Eng.

[128] Wahlbom CF, Eliasson A, Hahn-Hägerdal B (2001). Intracellular fluxes in a recombinant xylose-utilizing *Saccharomyces cerevisiae* cultivated anaerobically at different dilution rates and feed concentrations. Biotechnol Bioeng.

[129] Alff-Tuomala S, Salusjärvi L, Barth D, Oja M, Penttilä M, Pitkänen J-P (2016). Xylose-induced dynamic effects on metabolism and gene expression in engineered *Saccharomyces cerevisiae* in anaerobic glucose-xylose cultures. Appl Microbiol Biotechnol.

[130] Jin Y-S, Laplaza JM, Jeffries TW (2004). Saccharomyces cerevisiae engineered for xylose metabolism exhibits a respiratory response. Appl Environ Microbiol.

[131] Matsushika A, Goshima T, Hoshino T (2014). Transcription analysis of recombinant industrial and laboratory *Saccharomyces cerevisiae* strains reveals the molecular basis for fermentation of glucose and xylose. Microb Cell Factories..

[132] Salusjärvi L, Kankainen M, Soliymani R, Pitkänen J-P, Penttilä M, Ruohonen L (2008). Regulation of xylose metabolism in recombinant *Saccharomyces cerevisiae*. Microb Cell Factories..

[133] Feng X, Lian J, Zhao H (2015). Metabolic engineering of *Saccharomyces cerevisiae* to improve 1-hexadecanol production. Metab Eng.

[134] Brink DP, Borgström C, Tueros FG, Gorwa-Grauslund MF (2016). Real-time monitoring of the sugar sensing in *Saccharomyces cerevisiae* indicates endogenous mechanisms for xylose signaling. Microb Cell Factories.

[135] Eiteman MA, Ramalingam S (2015). Microbial production of lactic acid. Biotechnol Lett.

[136] Miller C, Fosmer A, Rush B, McMullin T, Beacom D, Suominen P. Industrial production of lactic acid. In: Comprehensive biotechnology. 2nd ed. Burlington: Academic Press; 2011. p. 179–88.

[137] Branduardi P, Sauer M, De Gioia L, Zampella G, Valli M, Mattanovich D (2006). Lactate production yield from engineered yeasts is dependent from the host background, the lactate dehydrogenase source and the lactate export. Microb Cell Factories..

[138] Ishida N, Saitoh S, Tokuhiro K, Nagamori E, Matsuyama T, Kitamoto K (2005). Efficient production of L-Lactic acid by metabolically engineered *Saccharomyces cerevisiae* with a genome-integrated L-lactate dehydrogenase gene. Appl Environ Microbiol.

[139] Skory CD (2003). Lactic acid production by *Saccharomyces cerevisiae* expressing a *Rhizopus oryzae* lactate dehydrogenase gene. J Ind Microbiol Biotechnol.

[140] Baek S-H, Kwon EY, Kim YH, Hahn J-S (2016). Metabolic engineering and adaptive evolution for efficient production of D-lactic acid in *Saccharomyces cerevisiae*. Appl Microbiol Biotechnol.

[141] Nielsen J (2014). Synthetic biology for engineering acetyl coenzymeA metabolism in yeast. mBio.

[142] Kim SJ, Seo SO, Park YC, Jin YS, Seo JH (2014). Production of butanediol from xylose by engineered *Saccharomyces cerevisiae*. J Biotechnol..

[143] Brat D, Boles E (2013). Isobutanol production from D-xylose by recombinant *Saccharomyces cerevisiae*. FEMS Yeast Res.

[144] Kim SJ, Seo SO, Jin YS, Seo JH (2013). Production of 2,3-butanediol by engineered *Saccharomyces cerevisiae*. Bioresour Technol.

[145] Brat D, Weber C, Lorenzen W, Bode HB, Boles E (2012). Cytosolic re-localization and optimization of valine synthesis and catabolism enables inseased isobutanol production with the yeast *Saccharomyces cerevisiae*. Biotechnol Biofuels.

[146] DeRisi JL, Iyer VR, Brown PO (1997). Exploring the metabolic and genetic control of gene expression on a genomic scale. Science.

[147] Egner A, Jakobs S, Hell SW (2002). Fast 100-nm resolution three-dimensional microscope reveals structural plasticity of mitochondria in live yeast. Proc Natl Acad Sci USA..

[148] Avalos JL, Fink GR, Stephanopoulos G (2013). Compartmentalization of metabolic pathways in yeast mitochondria improves the production of branched-chain alcohols. Nat Biotechnol.

[149] Sonderegger M, Jeppsson M, Hahn-Hägerdal B, Sauer U (2004). Molecular basis for anaerobic growth of *Saccharomyces cerevisiae* on xylose, investigated by global gene expression and metabolic flux analysis. Appl Environ Microbiol.

[150] Runquist D, Hahn-Hägerdal B, Bettiga M (2010). Increased ethanol productivity in xylose-utilizing *Saccharomyces cerevisiae* via a randomly mutagenized xylose reductase. Appl Environ Microbiol.

